# 

**Published:** 1914-12

**Authors:** 


### CONTENTS.

ORIGINAL COMMUNICATIONS—

Spinal Anesthesia in Genito-Urinary Surgery, by A. L. Fowler, M. D. .......................391

Report of Pellagra in the Georgia Baptist Orphan Home, by George C. Trimble, M. D. ....396

Meeting of the Fulton County Medical Society, by R. R. Daly, M. D. .......................402

EDITORIAL .......................................406

SELECTIONS AND ABSTRACTS ..................408

BOOK REVIEWS ....................................

